# Bubble-Point Pressure Deviations and Metastable Nucleation in Nanoconfined CO_2_–Shale Oil Systems: Effects of Temperature, Composition, Gas–Oil Ratio, and Pore-Scale Coupling

**DOI:** 10.3390/nano16191260

**Published:** 2026-10-06

**Authors:** Dongyan Qi, Jiawei Li, Qiang Liu, Shuang Wang, Siyuan Cui, Yue Lang, Jiayi Zhao, Xiaozhe Liang, Junjie Zhong

**Affiliations:** 1 Daqing Oilfield of China National Petroleum Cooperation, Daqing 163700, China; 2 State Key Laboratory of Continental Shale Oil, Daqing 163712, China; 3 State Key Laboratory of Deep Oil and Gas, School of Petroleum Engineering, China University of Petroleum (East China), Qingdao 266580, China

**Keywords:** shale oil, nanoconfinement, metastability, pore-scale connectivity, Peng-Robinson EOS, gas-oil ratio

## Abstract

Bubble-point behavior of shale oil under nanoconfinement is critical for understanding phase evolution during CO_2_-enhanced shale-oil recovery. In this study, visual micro/nanofluidic PVT experiments were conducted to systematically investigate bubble-point pressure deviations of pentane–CO_2_, hexadecane–CO_2_, pentane/decane/hexadecane–CO_2_, and shale oil–CO_2_ systems. Measurements were performed at gas–oil ratios of 130 and 260 and temperatures of 65–125 °C using a 10 nm single-scale pore model and a coupled 10 μm–10 nm pore-network model. Experimental results were compared with theoretical predictions from a confinement-modified Peng–Robinson equation of state incorporating capillary pressure, surface adsorption, and critical-property shifts. Furthermore, a metastable nucleation limit was introduced to describe the experimentally observed phase-transition pressure below the equilibrium bubble-point boundary. Pronounced downward deviations toward the metastable region were observed in isolated 10 nm pores. For the pentane–CO_2_ system at 65 °C and GOR = 130, the experimentally observed phase-transition pressure was approximately 3.5 MPa, compared with an equilibrium prediction of 4.3 MPa, corresponding to a relative deviation of 18.35%. The deviation decreased as temperature approached the critical region and was reduced at higher GOR. For example, at GOR = 260, the deviation of the hexadecane–CO_2_ system decreased from 40.63% at 65 °C to 10.01% at 125 °C. Fluid composition also strongly affected confinement sensitivity, with heavier hydrocarbon systems exhibiting stronger shifts toward metastable behavior. In contrast, bubble-point pressures in the coupled 10 μm–10 nm network remained close to the equilibrium predictions of the micrometer-scale pores and deviated significantly from the metastable limit. For the shale oil–CO_2_ system at 65 °C and GOR = 130, the relative deviation decreased from 29.77% in the isolated 10 nm pore model to only 2.43% in the coupled 10 μm–10 nm network. These results demonstrate that nanoconfined bubble-point behavior is governed by the coupled effects of thermodynamic conditions, fluid composition, metastability, and pore-scale connectivity.

## 1. Introduction

Shale oil has become an important unconventional petroleum resource, but its efficient development remains challenging because hydrocarbons are hosted in complex micro- and nanoscale pore systems and commonly exhibit limited natural mobility [1,2]. Carbon dioxide (CO_2_) injection has attracted considerable attention for shale-oil enhanced recovery because CO_2_ can dissolve into crude oil, reduce oil viscosity, promote oil swelling, extract light and intermediate hydrocarbons, and improve fluid mobility under suitable pressure and temperature conditions [3,4,5]. During CO_2_ injection and subsequent pressure depletion, the phase behavior of the CO_2_–oil mixture directly controls gas liberation, fluid expansion, compositional redistribution, and the transition between single-phase and two-phase flow. Among the relevant phase-equilibrium parameters, bubble-point pressure is particularly important because it defines the onset of gas evolution from the liquid phase and therefore influences relative permeability, flow resistance, and production performance [6,7,8]. Conventional phase-behavior analysis is generally based on bulk-fluid thermodynamics and equation-of-state calculations. However, shale oil is largely confined within pores whose characteristic dimensions can approach the molecular scale, where fluid-wall interactions and confinement effects may cause the observed phase behavior to deviate from bulk predictions [9,10]. Therefore, accurately determining the bubble-point behavior of CO_2_–shale-oil systems under nanoscale confinement is essential for understanding fluid evolution during CO_2_ injection and for improving phase-state descriptions used in shale-oil reservoir development.

Previous studies have shown that nanoconfinement can alter the thermodynamic properties and phase boundaries of hydrocarbon mixtures, including critical properties, saturation pressures, density, and phase-transition behavior [11]. These effects have been investigated using molecular simulation, modified equations of state, adsorption-based thermodynamic models, and micro/nanofluidic visualization [12]. Most available studies, however, focus on idealized binary mixtures, individual hydrocarbon components, or isolated nanopores, whereas actual shale reservoirs contain highly heterogeneous pore systems in which nanoscale matrix pores are connected with larger pores, microfractures, and hydraulic fractures [13]. Under such conditions, phase behavior may be affected not only by the local confinement scale but also by mass exchange and thermodynamic communication among pores of different sizes [14]. Consequently, phase-transition behavior observed in a single nanopore may not directly represent that of a connected multiscale pore network. In addition, temperature and overall CO_2_ content can shift a fluid system toward or away from its critical region, which may change its sensitivity to nanoscale confinement [15]. Although theoretical studies have predicted such dependencies, systematic experimental evidence across different temperatures, gas–oil ratios, and pore-scale configurations remains limited. For example, Cho et al. measured the bubble-point pressures of methane–hydrocarbon mixtures in approximately 4 nm mesoporous media and observed lower bubble-point pressures than those of the corresponding bulk fluids [8]. Zhong et al. investigated connected channels spanning four orders of magnitude from 10 nm to 10 μm and reported an approximately threefold suppression of the bubble-point pressure under nanoconfinement [11]. In particular, direct comparisons between a single 10 nm pore environment and a coupled micro–nanopore network are scarce [16]. Clarifying whether multiscale pore coupling enhances, suppresses, or redistributes the apparent nanoconfinement effect is therefore necessary for establishing a more realistic description of CO_2_–oil phase behavior in shale reservoirs.

Fluid composition introduces an additional level of complexity because shale oil contains hydrocarbons with a broad molecular-weight distribution and light, intermediate, and heavy components may respond differently to nanoscale confinement and CO_2_ dissolution [17,18]. Light hydrocarbons generally exhibit higher volatility and stronger affinity for the CO_2_-rich phase, whereas heavier hydrocarbons have larger molecular sizes and lower volatility and may be more strongly affected by fluid–wall interactions and spatially restricted molecular organization in nanoscale pores [12]. As a result, the bubble-point shift of a CO_2_–oil mixture may depend strongly on the relative proportions of light, intermediate, and heavy components rather than on pore size alone. However, the compositional contribution to nanoconfined bubble-point deviation has not been systematically isolated using compositionally controlled model fluids and then compared with real shale oil [19]. Xu et al. [20] recently investigated the vaporization of a methane/propane mixture in 50 and 1000 nm pores and showed that the experimentally observed phase-transition pressure shifts toward the metastable nucleation limit under lower temperatures and stronger confinement. Building on this work, the present study extends the investigation to CO_2_–oil systems and directly compares an isolated 10 nm pore with a coupled 10 μm–10 nm pore network, with particular emphasis on the role of pore-scale connectivity.

Building on previous studies, the present study investigates four representative CO_2_–oil systems: pentane–CO_2_, hexadecane–CO_2_, pentane/decane/hexadecane–CO_2_, and shale oil–CO_2_. Bubble-point pressures are measured at gas–oil ratios of 130 and 260 and at temperatures of 65, 85, 105, and 125 °C using both an isolated 10 nm pore model and a coupled 10 μm–10 nm pore-network model. The objectives of this study are to quantify the effects of temperature, fluid composition, gas-oil ratio, and pore-scale configuration on the deviation between experimentally observed phase-transition pressures and theoretical equilibrium predictions; to clarify the role of metastable nucleation in these deviations; and to determine how pore-scale connectivity modifies the apparent nanoconfinement effect. The scientific novelty of this work lies in the direct experimental comparison between isolated 10 nm confinement and a coupled 10 μm–10 nm pore network under comparable thermodynamic and compositional conditions, thereby enabling the effects of isolated nanoconfinement and micro-nanopore coupling on phase-transition behavior to be distinguished.

## 2. Methods

### 2.1. Experimental Setup and Procedures

**Figure 1:**
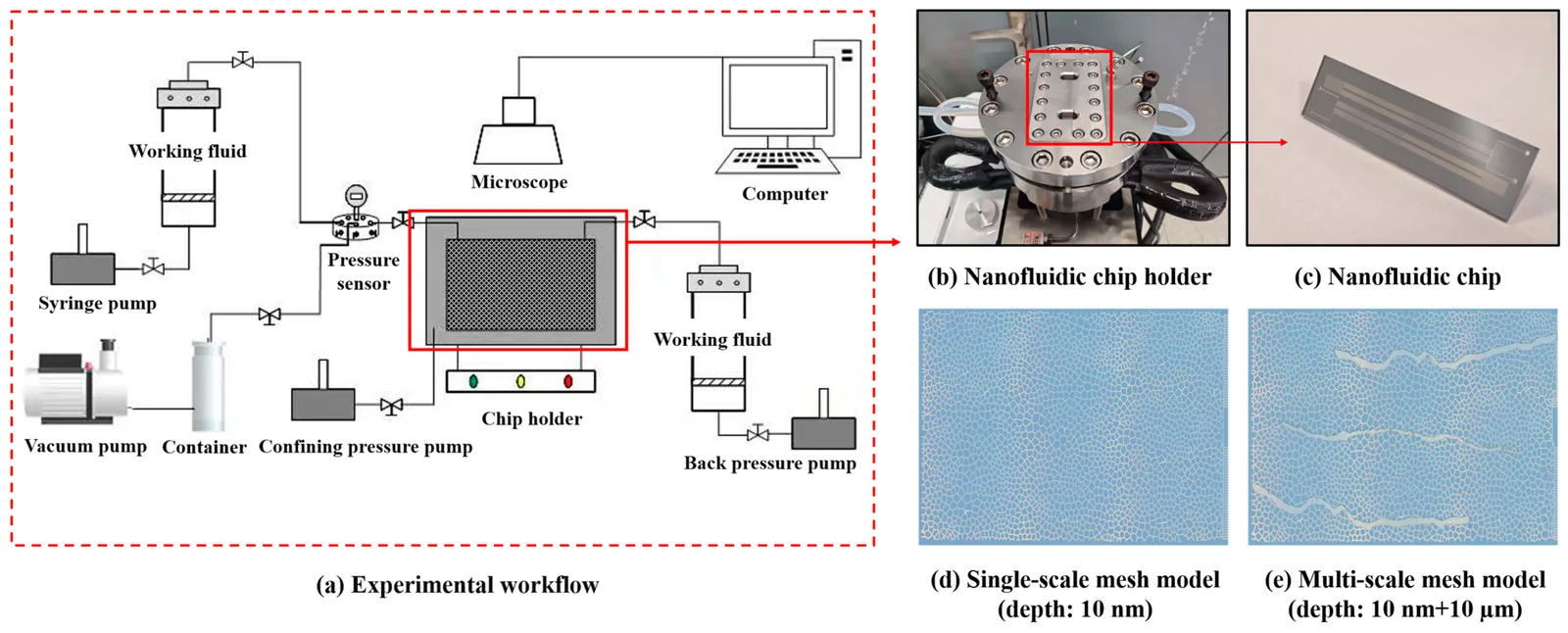
Experimental setup and nanofluidic pore models used for bubble-point measurements: (**a**) schematic of experimental workflow; (**b**) high-pressure chip holder; (**c**) nanofluidic chip; (**d**) 10 nm single-scale pore model; and (**e**) coupled 10 μm–10 nm multiscale pore model.

A visual PVT system (Nantong Xinhuacheng Scientific Research Instrument Co., Ltd., Haian, Jiangsu, China) integrated with micro/nanofluidic chips was developed to determine the bubble-point pressures of CO_2_–oil mixtures under confined conditions. As illustrated in Figure 1, the experimental platform mainly consisted of a fluid injection and pressure-control system, a micro/nanofluidic chip and high-pressure chip holder, a temperature-control system, a pressure-monitoring system, and an optical visualization and image-recording system. The working fluids were stored in high-pressure containers and introduced into the chip through a syringe pump. A back-pressure pump was connected to the outlet side to regulate the system pressure, while a pressure sensor was positioned close to the chip to continuously monitor the pressure during the experiment. The pressure sensor had a measurement accuracy of ±0.01 MPa. Before fluid injection, a vacuum pump was used to evacuate the chip and connecting pipelines. A confining-pressure pump provided external pressure to the chip holder to ensure reliable sealing and mechanical stability under elevated pressure. The phase behavior inside the pore network was continuously monitored using an optical microscope, with an optical resolution of approximately 1 μm, and the recorded images were transferred to a computer for real-time observation and subsequent analysis.

Two types of pore models were employed to distinguish the phase behavior under isolated nanoconfinement from that in a multiscale pore network. The first was a single-scale pore model with a characteristic confinement dimension of 10 nm, representing strongly confined nanopores in shale. The 10 nm scale was selected as a representative strongly confined pore size relevant to shale matrix pores and to provide a clear nanoscale contrast with the 10 μm region in the coupled pore model. It should not be interpreted as a unique or critical pore size at which metastable behavior occurs. Rather, the present experiments are intended to reveal the phase-transition characteristics under a representative strong-confinement condition, while the systematic dependence on pore widths below and above 10 nm requires further investigation. The second consisted of interconnected 10 μm and 10 nm pore regions, representing a coupled micro-nanopore network in which nanoscale pores communicate with larger pore spaces. The actual depth of the 10 nm nanochannels was characterized by atomic force microscopy (AFM), and the corresponding surface topography and depth profile are provided in Figure S1 of the Supporting Information. The corresponding pore-network structures and the experimental chip are shown in Figure 1. This design enabled direct comparison between phase transitions occurring in an isolated nanoscale environment and those occurring in a connected multiscale pore system.

Four representative CO_2_–oil systems were investigated: pentane–CO_2_, hexadecane–CO_2_, pentane/decane/hexadecane–CO_2_, and shale oil–CO_2_. The purity of pentane, decane, hexadecane, and CO_2_ used in the experiments were all greater than 99.5%. Pentane and hexadecane were selected as representative light and heavy hydrocarbon components, respectively, while the pentane/decane/hexadecane mixture was used as a simplified multicomponent fluid containing light, intermediate, and heavy fractions. The shale oil sample was obtained from the Gulong shale-oil reservoir in Daqing Oilfield, China. To characterize the compositional distribution of the real shale oil, whole-oil carbon-number analysis was conducted, and the resulting carbon-number distribution based on mass fraction is summarized in Table 1. Based on this distribution, the mass fractions of light, intermediate, and heavy hydrocarbons in Gulong shale oil were determined and used to define the relative proportions of pentane, decane, and hexadecane, respectively, in the simplified model fluid.

Experiments were performed at gas-oil ratios (GORs) of 130 and 260 and at temperatures of 65, 85, 105, and 125 °C. Both the 10 nm single-scale pore model and the coupled 10 μm–10 nm pore-network model were used for each fluid system. The combination of controlled fluid compositions, thermodynamic conditions, and pore configurations provided a systematic experimental matrix for evaluating the effects of temperature, composition, GOR, and pore-scale coupling on confined bubble-point behavior.

Prior to each experiment, the entire fluid pathway, including the micro/nanofluidic chip, high-pressure tubing, valves, and fluid containers, was assembled and subjected to a leak test. The chip and connecting lines were subsequently evacuated to remove residual gas and minimize contamination from the preceding experiment. The prepared working fluid was then introduced into the micro/nanofluidic chip using the syringe pump until the pore network was completely saturated. The system pressure was increased above the expected bubble-point pressure to ensure that the fluid inside the chip remained in a stable single-liquid-phase state.

After fluid saturation, the temperature-control system was adjusted to the target experimental temperature of 65, 85, 105, or 125 °C. The system was maintained at the prescribed temperature and elevated pressure until thermal and pressure equilibrium were reached. During this period, the microscopic field of view was continuously inspected to confirm the absence of a gas phase within the pore network. Once a stable initial state had been established, depressurization was initiated. The system pressure was reduced stepwise while the temperature was maintained constant. The depressurization rate was controlled at 0.1 MPa/min, and the system was maintained for 5 min after each pressure reduction to ensure pressure stabilization and phase response before the next pressure step. In this study, the pressure at which a stable gas phase was first observed after the 5 min stabilization period was defined as the experimentally observed metastable nucleation pressure and is referred to as the experimental bubble-point pressure. This pressure is distinguished from the thermodynamic equilibrium bubble-point pressure predicted by the PR-EOS and the theoretical metastable nucleation limit calculated from the nucleation model. If no stable gas phase appeared during the 5 min stabilization period, the pressure was further reduced. Once stable vapor nucleation occurred, the gas phase persisted and continuously grew until it became optically distinguishable. Therefore, the identification of the experimental nucleation pressure does not depend on resolving the initial size of the vapor nucleus itself. The 5 min stabilization period also excludes transient optical fluctuations and ensures that the recorded pressure corresponds to stable vapor nucleation. Consequently, the finite optical resolution is not considered the primary cause of the systematic pressure deviation observed in this study. The pressure variation was continuously recorded, and the pore network was simultaneously monitored through the microscope.

The onset of vaporization was identified from the first appearance of a stable and distinguishable gas phase within the observation region. Transient optical fluctuations or isolated features that disappeared immediately were not considered evidence of phase transition. The system pressure corresponding to the persistent appearance of the gas phase was recorded as the experimental bubble-point pressure. After each measurement, the pressure was increased above the bubble point to restore the fluid to a single-phase state before the next measurement was performed.

The same procedure was applied to the 10 nm single-scale and 10 μm–10 nm coupled pore models. For each fluid composition, measurements were conducted at GORs of 130 and 260 over the four investigated temperatures. Each experimental condition was independently repeated three times, and the reported phase-transition pressures represent the average values of the three measurements. The standard deviations of the repeated measurements did not exceed 0.3 MPa, indicating good experimental reproducibility. The resulting experimental bubble points were subsequently compared with the calculated confined phase boundaries to quantify the deviation between experimentally observed vaporization and thermodynamic predictions.

### 2.2. Fluid Bubble-Point Prediction Under Nanoconfinement

The Peng-Robinson equation of state (PR-EOS) was adopted as the fundamental thermodynamic model for calculating the phase behavior of the investigated CO_2_–oil systems. For a homogeneous bulk fluid, the PR-EOS is expressed as follows [20]:

(1) $$P=\frac{RT}{V-b}-\frac{a\alpha (T)}{V(V+b)+b(V-b)}$$

where *P* and *T* denote pressure and temperature, respectively; *R* is the universal gas constant; *V* is molar volume; and a and b characterize the attractive and repulsive intermolecular interactions, respectively. For each pure component,

(2) $$a=0.45724\frac{R^{2}T_{c}^{2}}{P_{c}}$$

(3) $$b=0.07780\frac{RT_{c}}{P_{c}}$$

(4) $$\alpha (T)={\left[1+(0.37464+1.54226)\omega -0.26992\omega^{2}(1-\sqrt{\frac{T}{T_{c}}})\right]}^{2}$$

where *T_c_*, *P_c_*, and ω are the critical temperature, critical pressure, and acentric factor, respectively.

For multicomponent mixtures, conventional mixing rules were used:

(5) $$a=\sum_{i}\sum_{j}x_{i}x_{j}a_{ij}$$

(6) $$b=\sum_{i}x_{i}b_{i}$$

(7) $$a_{ij}=(1-k_{ij})\sqrt{a_{i}a_{j}}$$

where *x_i_* and *x_j_* are liquid-phase mole fractions and *k_ij_* represents the binary interaction parameter between components i and j.

The conventional PR-EOS describes phase equilibrium under bulk conditions. When the characteristic pore size decreases to the nanoscale, however, the large surface-to-volume ratio and strong geometric confinement introduce additional effects that are not explicitly included in the bulk EOS. In the present study, the PR-EOS was therefore extended by considering three major confinement-related contributions: capillary pressure across the curved vapor-liquid interface, surface adsorption near the pore wall, and confinement-induced shifts in critical properties. These corrections were incorporated into the phase-equilibrium calculations to obtain the theoretical bubble-point curves under nanoconfinement.

When vapor and liquid coexist in a confined pore, the curved vapor-liquid interface results in a pressure difference between the two phases. This pressure difference becomes increasingly important as the pore dimension decreases and the interface curvature increases. The capillary pressure is defined as

(8) $$P_{cap}=P_{V}-P_{L}$$

where *P_cap_* is the capillary pressure, *P_V_* is the vapor-phase pressure, and *P_L_* is the liquid-phase pressure.

The pressure difference across the vapor-liquid interface is calculated using the Young-Laplace equation:

(9) $$P_{cap}=\sigma (\frac{1}{R_{1}}+\frac{1}{R_{2}})$$

where *σ* is the vapor-liquid interfacial tension, and *R_1_* and *R_2_* are the two principal radii of curvature of the interface.

For the rectangular micro/nanochannels considered in this study, the two curvature radii are related to the channel geometry and surface wettability. After accounting for the adsorption layer near the pore wall, they are expressed as

(10) $$R_{1}=(\frac{W}{2}-\delta )/\cos\theta$$

(11) $$R_{2}=(\frac{H}{2}-\delta )/\cos\theta$$

where *W* and *H* are the channel width and depth, respectively; *δ* is the thickness of the adsorbed layer; and *θ* is the contact angle between the confined fluid and the pore wall. The oil-gas interfacial contact angle was estimated directly from the in situ microscopic images obtained during the microfluidic phase-transition experiments. The angle between the tangent to the oil-gas meniscus and the channel wall was approximately 45°, and this value was used in the capillary-pressure calculations.

Substitution of Equations (10) and (11) into Equation (9) gives

(12) $$P_{cap}=\sigma \cos\theta (\frac{1}{\frac{W}{2}-\delta }+\frac{1}{\frac{H}{2}-\delta })$$

Equation (12) indicates that the capillary-pressure contribution depends simultaneously on pore geometry, surface wettability, adsorption-layer thickness, and vapor-liquid interfacial tension.

The interfacial tension of a multicomponent fluid was evaluated using the Macleod-Sugden relationship:

(13) $$\sigma ={\left(\rho_{L}\sum_{i=1}x_{i}P_{chi}-\rho_{V}\sum_{i=1}y_{i}P_{chi}\right)}^{4}$$

where *ρ_L_* and *ρ_V_* are the molar densities of the liquid and vapor phases, respectively; *x_i_* and *y_i_* are the mole fractions of component i in the liquid and vapor phases; and *P_chi_* is the Parachor parameter of component i.

The liquid- and vapor-phase densities and compositions used in Equation (13) were obtained from the phase-equilibrium calculation. Therefore, the interfacial tension was updated together with the phase compositions during the iterative calculation rather than being treated as a constant value.

With the capillary-pressure correction, the pressures of the vapor and liquid phases satisfy

(14) $$P_{V}=P_{L}+P_{cap}$$

Thus, unlike bulk vapor-liquid equilibrium calculations in which both phases are normally assumed to be at the same pressure, the confined phase-equilibrium calculation allows different vapor- and liquid-phase pressures connected through the capillary pressure.

Under nanoconfinement, strong fluid–wall interactions lead to the formation of an adsorbed layer near the pore surface, reducing the effective pore space available to the mobile fluid. The adsorption-layer thickness, δ, was estimated using a pore-size- and molecular-weight-dependent correlation reported in previous studies [20]. For the 10 nm nanochannels, the calculated adsorption-layer thicknesses for CO_2_, n-pentane, n-decane, and n-hexadecane were 0.357, 0.664, 1.062, and 0.839 nm, respectively. Accordingly, the effective pore radius was calculated as

(15) $$r_{e}=r_{p}+\delta$$

where *r_p_* is the nominal pore radius and *r_e_* is the effective pore radius after accounting for surface adsorption. For the slit-like nanochannels used in this study, *r_p_* was taken as half of the channel depth.

Surface adsorption also changes the effective molar volume of the mobile fluid. The adsorption fraction, *χ*, is defined as the ratio of adsorbed molecules to the total number of molecules in the pore. The adsorption-corrected molar volume is therefore expressed as

(16) $$V_{m}=V/(1-\chi )$$

where *V* is the original molar volume and *V_m_* is the effective molar volume after adsorption correction. The corrected *V_m_* was subsequently introduced into the PR-EOS to account for the influence of surface adsorption on the confined phase behavior.

Nanoconfinement also causes the critical properties of the confined fluid to deviate from their bulk values. This effect becomes increasingly pronounced when the pore dimension approaches the characteristic molecular size. In this study, the confinement-induced critical-temperature shift was correlated with the ratio of the effective pore radius to the Lennard-Jones molecular-size parameter:

(17) $$\Delta T_{c}=0.8594\times {\left(\frac{r_{e}}{\sigma_{LJ}}\right)}^{-1}$$

where *ΔT_c_* is the relative critical-temperature shift, and *σ_LJ_* is the Lennard-Jones size parameter.

The corresponding relative critical-pressure shift was calculated as

(18) $$\Delta P_{c}=1.7188\times {\left(\frac{r_{e}}{\sigma_{LJ}}\right)}^{-1}-0.7386\times {\left(\frac{r_{e}}{\sigma_{LJ}}\right)}^{-2}$$

where *ΔP_c_* denotes the relative critical-pressure shift. The Lennard-Jones size parameter was calculated from the bulk critical properties of each component according to $\sigma_{LJ}=0.244\times {\left(\frac{T_{c}}{P_{c}}\right)}^{1/3}$; the calculated values for CO_2_, n-pentane, n-decane, and n-hexadecane were 0.393, 0.590, 0.755, and 0.912 nm, respectively [21]. The confinement-corrected critical temperature and pressure obtained from these relationships were subsequently used to update the PR-EOS parameters for bubble-point calculations. In this way, the influence of pore size relative to molecular size was incorporated into the prediction of confined phase behavior.

The equilibrium constants, phase compositions, densities, interfacial tension, and capillary pressure were iteratively updated until the phase-equilibrium convergence criterion was satisfied. The resulting liquid-phase pressure was taken as the theoretical equilibrium bubble-point pressure *P_sat_* at the specified temperature and confinement condition.

However, the experimentally observed bubble-point pressure may deviate from the equilibrium value because the appearance of a vapor phase requires the formation of a stable vapor nucleus and the overcoming of an interfacial free-energy barrier. Therefore, the metastable limit pressure was further considered based on classical nucleation theory. Following the Furth approximation for metastable limits [22], the pressure difference between the equilibrium bubble point and the metastable limit can be expressed as

(19) $$P_{sp}(T_{1})=P_{sat}(T_{1})-C\gamma_{2}^{3/2}{\left(k_{B}T_{1}\right)}^{-1/2}$$

where *P_sp_*(*T*_1_) represents the metastable limit pressure at temperature T_1_, *P_sat_*(*T*_1_) is the thermodynamic equilibrium bubble-point pressure, *C* is the metastable-limit coefficient, $\gamma_{2}^{3/2}$ is the vapor-liquid interfacial tension, and *k_B_* is the Boltzmann constant. Following the Furth formulation for the metastable limit adopted by Bao et al. [23], the coefficient C was set to 1.32. According to this relationship, the metastable limit pressure is lower than the equilibrium bubble-point pressure due to the additional pressure undersaturation required for vapor nucleation. The calculated metastable pressure provides a theoretical estimation of the experimentally observed phase-transition pressure, particularly for confined systems where interfacial effects become significant. Repeating the calculation over the investigated temperature range generated the theoretical equilibrium and metastable bubble-point curves used for comparison with the experimental measurements.

## 3. Results and Discussion

All experimental phase-transition pressures reported in the following discussion represent the mean values of three independent measurements, with standard deviations not exceeding 0.3 MPa. The percentage deviations were calculated using these mean experimental values. For the following discussion, the experimentally measured bubble-point pressure was evaluated by comparing it with both the equilibrium bubble-point pressure and the metastable nucleation limit predicted by the theoretical model. The equilibrium bubble-point pressure represents the thermodynamic phase-transition condition calculated using the confinement-modified PR-EOS, while the metastable nucleation limit represents the pressure boundary associated with vapor formation under interfacial free-energy constraints. Therefore, the experimentally observed bubble-point pressure is expected to be located between these two theoretical limits. For the 10 nm single-scale pore model, the experimental bubble-point pressure was compared with the theoretical prediction under nanoscale confinement. For the coupled 10 μm–10 nm pore-network model, the experimental results were compared with the theoretical bubble-point prediction corresponding to the micrometer-scale pore domain.

### 3.1. Effect of Temperature on Bubble-Point Deviation

**Figure 2:**
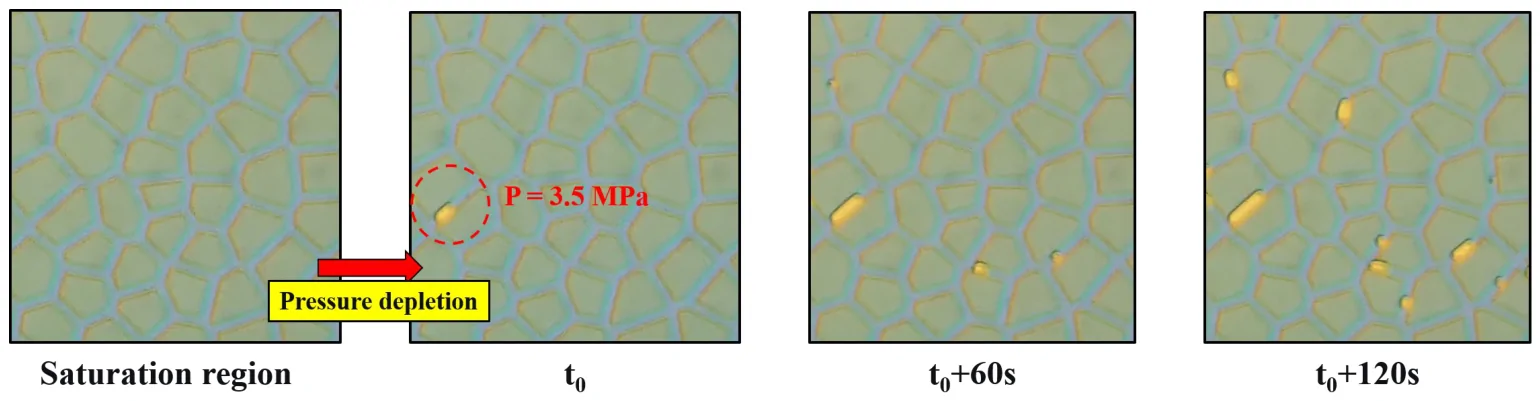
Bubble nucleation of pentane–CO_2_ system (GOR = 130) in 10 nm-deep nanochannels at 65 °C, t_0_ denotes onset of bubble nucleation.

Temperature exerted a pronounced influence on the deviation between the experimentally observed and theoretically predicted bubble-point pressures under nanoconfinement. Figure 2 shows the pressure-dependent phase-transition process of the pentane–CO_2_ system during pressure depletion at 65 °C and GOR = 130 in the 10 nm pore model. Initially, the confined fluid remained in a homogeneous single-phase state. With continuous pressure reduction, no visible gas phase appeared until the system approached the phase-transition region. Subsequently, stable gas bubbles emerged and gradually expanded within the nanopore. The pressure corresponding to the first persistent appearance of the vapor phase was identified as the experimentally determined bubble-point pressure.

At 65 °C, the first stable gas bubbles appeared at approximately 3.5 MPa, whereas the theoretical equilibrium bubble-point pressure was 4.3 MPa, corresponding to a relative deviation of 18.35%. As the temperature increased to 85 °C, the experimental bubble point increased to 5.0 MPa, and the deviation decreased to 4.35%. At 105 °C, the measured bubble point further increased to 5.6 MPa and approached the theoretical value of 5.7 MPa, with a deviation of only 0.69%. At 125 °C, the pentane–CO_2_ system entered the supercritical region and no distinct bubble point could be identified.

The same temperature dependence was observed for the other fluid systems. For example, at GOR = 260 and in the 10 nm pore model, the relative deviation of the hexadecane–CO_2_ system decreased from 40.63% at 65 °C to 27.61%, 18.00%, and 10.01% at 85, 105, and 125 °C, respectively. The pentane/decane/hexadecane–CO_2_ system showed a similar decrease from 24.30% at 65 °C to 1.57% at 125 °C, while the deviation of the shale oil–CO_2_ system decreased from 25.25% to 6.94% over the same temperature range. Overall, the deviation between the theoretical equilibrium bubble-point predictions and the experimental measurements was further quantified using the root-mean-square error (RMSE) and mean absolute percentage error (MAPE), which were 2.19 MPa and 11.45%, respectively.

The magnitude of the bubble-point suppression observed in this study is comparable to previous nanoconfinement measurements. Cho et al. [8] reported bubble-point reductions of approximately 9.16–35.28% for methane-decane mixtures and 12.66–29.48% for methane-octane mixtures confined in approximately 3.5–3.7 nm mesoporous media. In comparison, the present experiments in 10 nm nanochannels show deviations ranging from less than 1% to approximately 40%, depending on temperature, fluid composition, and GOR. Zuo et al. [10] also predicted a progressive decrease in shale-oil bubble-point pressure with decreasing pore size, further supporting the strong pore-size dependence of confined phase behavior.

This behavior can be interpreted from the temperature dependence of vapor-liquid interfacial properties and metastable phase-transition behavior. At relatively low temperatures, the larger differences in density and composition between the liquid and vapor phases result in higher vapor-liquid interfacial tension, increasing the resistance to the formation of an observable vapor phase. Consequently, gas evolution occurs only after the pressure decreases below the equilibrium bubble-point pressure, leading to a larger downward deviation in the experimentally determined bubble point.

**Figure 3:**
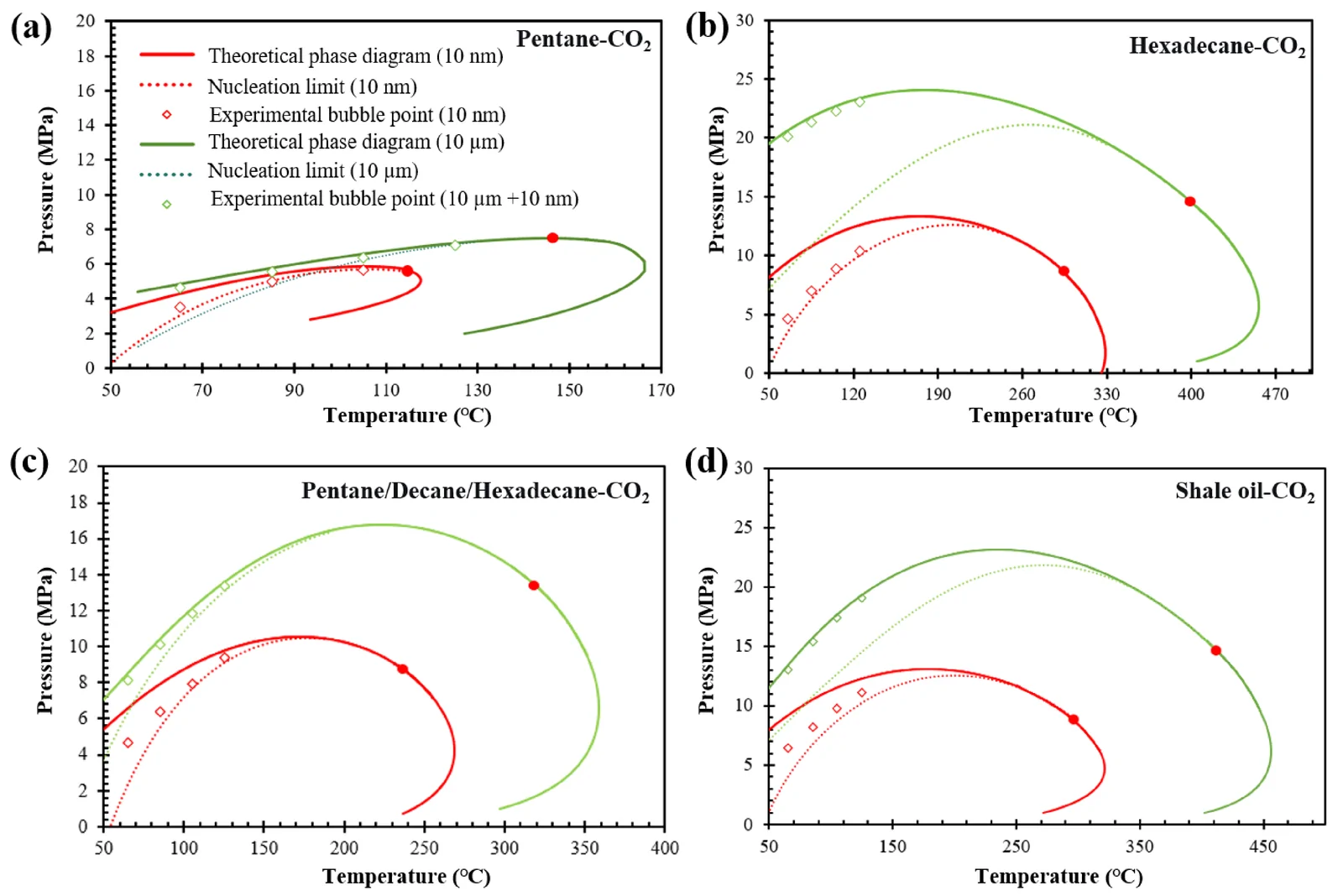
Bubble-point of nanoconfined CO_2_–oil systems at GOR = 130: comparison between equilibrium predictions, nucleation limits, and experimental measurements. (**a**) Pentane–CO_2_ system; (**b**) hexadecane–CO_2_ system; (**c**) pentane/decane/hexadecane–CO_2_ system; and (**d**) shale oil–CO_2_ system. The legend shown in panel (**a**) is applied to all panels.

The comparison between experimental bubble points and metastable nucleation limits further demonstrates this temperature dependence. As shown in Figure 3, under lower-temperature conditions, the experimental bubble points tend to approach the metastable nucleation limit rather than the equilibrium bubble-point curve. This indicates that the experimentally observed phase transition is influenced not only by equilibrium thermodynamics but also by the pressure requirement for vapor nucleation. The proximity of the experimental pressures to the metastable nucleation limit is therefore interpreted as evidence supporting a significant contribution of metastable vapor nucleation under 10 nm confinement, rather than as conclusive proof of a unique nucleation mechanism. The present results establish this behavior for the investigated 10 nm confinement condition; whether the magnitude of the deviation follows the same trend at smaller or larger pore widths remains to be systematically examined. As temperature increases toward the critical region, the differences between the liquid and vapor phases gradually diminish, accompanied by reduced interfacial resistance and weakened metastable behavior. Therefore, the experimentally measured bubble-point pressure progressively approaches the equilibrium bubble-point pressure.

The temperature effect is particularly significant in the strongly confined 10 nm pore model, where the large surface-to-volume ratio enhances the influence of interfacial effects. These results demonstrate that the experimentally observed bubble point under nanoconfinement represents a transition state between equilibrium phase behavior and metastable nucleation behavior.

### 3.2. Effect of Fluid Composition on Bubble-Point Deviation

Fluid composition strongly affects the magnitude of bubble-point deviation under nanoconfinement. Figure 3 compares the equilibrium bubble-point curves, metastable nucleation limits, and experimental measurements of different CO_2_–oil systems at GOR = 130.

All investigated fluid systems exhibit downward deviations from the equilibrium bubble-point predictions in the 10 nm pore model, but the magnitude of the deviation varies significantly with fluid composition. At 105 °C and GOR = 130, the experimentally measured bubble-point pressures were approximately 5.6 MPa for the pentane–CO_2_ system, 7.9 MPa for the pentane/decane/hexadecane–CO_2_ system, 9.8 MPa for the shale oil–CO_2_ system, and 8.9 MPa for the hexadecane–CO_2_ system. Although the absolute bubble-point pressure depends on the overall phase behavior of each mixture, the deviation relative to the corresponding theoretical prediction reveals a clear compositional dependence.

At 105 °C and GOR = 130, the pentane–CO_2_ system exhibited the smallest relative deviation, approximately 0.69%. The deviation increased to about 12% for the pentane/decane/hexadecane–CO_2_ system and 14.93% for the shale oil–CO_2_ system, while the hexadecane–CO_2_ system showed the largest deviation, reaching approximately 25%. These results indicate that the sensitivity of bubble-point behavior to nanoconfinement becomes progressively stronger for heavier hydrocarbon systems.

The comparison with metastable nucleation limits further reveals the role of composition in controlling the phase-transition pathway. The experimental bubble points of the pentane–CO_2_ system remain close to the equilibrium bubble-point curves, indicating relatively weak metastable effects. In contrast, the hexadecane–CO_2_ system exhibits a stronger downward shift and approaches the metastable nucleation limit. The pentane/decane/hexadecane–CO_2_ and shale oil–CO_2_ systems show intermediate behavior, suggesting that increasing hydrocarbon complexity and heaviness enhances the tendency of the confined fluid to undergo delayed vapor formation.

**Figure 4:**
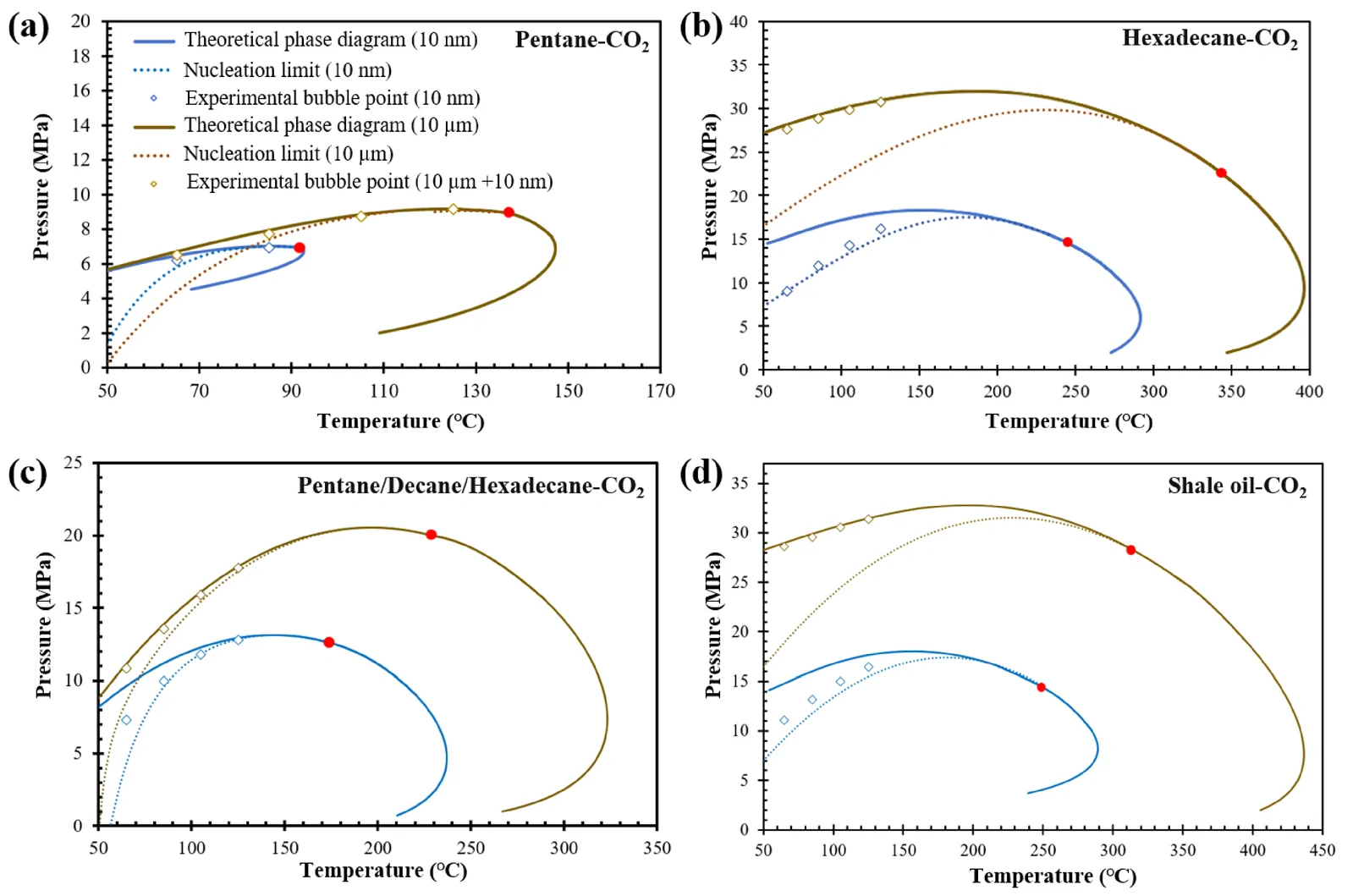
Bubble-point of nanoconfined CO_2_–oil systems at GOR = 260: comparison between equilibrium predictions, nucleation limits, and experimental measurements. (**a**) Pentane–CO_2_ system; (**b**) hexadecane–CO_2_ system; (**c**) pentane/decane/hexadecane–CO_2_ system; and (**d**) shale oil–CO_2_ system. The legend shown in panel (**a**) is applied to all panels.

The composition-dependent trend remains consistent under the higher GOR condition. As shown in Figure 4, the experimental bubble points of different fluid systems at GOR = 260 remain below the equilibrium bubble-point curves, while their positions relative to the metastable limits vary with composition.

According to Table 2, at 65 °C and GOR = 260, the relative deviations of the pentane–CO_2_, pentane/decane/hexadecane–CO_2_, shale oil–CO_2_, and hexadecane–CO_2_ systems are 4.10%, 24.30%, 25.25%, and 40.63%, respectively. This confirms that the heavier hydrocarbon systems exhibit stronger sensitivity to nanoscale confinement.

The enhanced deviation of heavier systems is likely associated with several coupled thermodynamic effects. Heavier hydrocarbons generally possess lower volatility and larger molecular dimensions, which can increase their sensitivity to restricted molecular organization and fluid–wall interactions within nanoscale pores. In addition, heavier-component-rich systems maintain larger differences in vapor-liquid properties under the investigated conditions, resulting in stronger interfacial effects during vapor formation. Therefore, the stronger confinement sensitivity of heavier fluids originates from the combined effects of molecular size, volatility, interfacial properties, and the distance from the critical region.

### 3.3. Effect of Gas-Oil Ratio on Bubble-Point Deviation

The influence of gas-oil ratio (GOR) on bubble-point deviation was evaluated by comparing the experimental results obtained at GOR = 130 and 260 under identical temperature, composition, and pore-scale conditions. Figure 3 and Figure 4 present the equilibrium bubble-point curves, metastable nucleation limits, and experimental bubble-point pressures at the two GOR conditions, respectively.

Increasing GOR systematically reduces the relative deviation between the experimentally observed bubble-point pressure and the equilibrium prediction in the 10 nm pore model. At the same temperature and fluid composition, the experimental bubble points obtained at GOR = 260 are generally closer to the equilibrium bubble-point curves and farther away from the metastable nucleation limits compared with those at GOR = 130. This indicates that increasing CO_2_ content weakens the metastable characteristics of the confined fluid and reduces the pressure shift required for observable vapor formation.

For example, at 105 °C, the relative deviation of the hexadecane–CO_2_ system decreases from approximately 25% at GOR = 130 to 18.00% at GOR = 260. Similarly, the deviation of the pentane/decane/hexadecane–CO_2_ system decreases from approximately 12% to 8.00%, while that of the shale oil–CO_2_ system decreases from 14.93% to 11.93%. These results demonstrate that increasing GOR can effectively reduce the influence of nanoconfinement on experimentally determined bubble-point pressure.

The shale oil–CO_2_ system provides a representative example of the GOR effect over the entire investigated temperature range. As summarized in Table 3, in the 10 nm single-scale model, increasing GOR from 130 to 260 reduces the relative deviation from 29.77% to 25.25% at 65 °C, from 21.51% to 17.92% at 85 °C, from 14.93% to 11.93% at 105 °C, and from 9.65% to 6.94% at 125 °C. A similar trend is observed in the coupled 10 μm–10 nm pore network, where the deviations are significantly smaller but still decrease with increasing GOR.

The reduction in bubble-point deviation with increasing GOR can be attributed to the change in the thermodynamic state of the CO_2_–oil mixture. A higher GOR increases the CO_2_ fraction in the mixture, shifting the overall composition toward a CO_2_-rich condition and moving the system closer to the critical region under the investigated temperature range. As the system approaches the critical region, the differences in density and composition between the liquid and vapor phases become smaller, resulting in reduced vapor-liquid interfacial tension and weaker resistance to phase transition.

The comparison with metastable nucleation limits further supports this interpretation. Under lower GOR conditions, the experimental bubble points are closer to the metastable nucleation limits, indicating that hydrocarbon-rich mixtures require a larger pressure reduction before stable vapor formation occurs. In contrast, higher GOR conditions reduce the distance between the experimental bubble point and the equilibrium phase boundary because the weakened interfacial effects facilitate vapor formation. Therefore, GOR influences not only the equilibrium phase envelope of CO_2_–oil mixtures but also the degree of metastable behavior during nanoscale phase transition.

### 3.4. Effect of Pore-Scale Coupling on Bubble-Point Deviation

**Figure 5:**
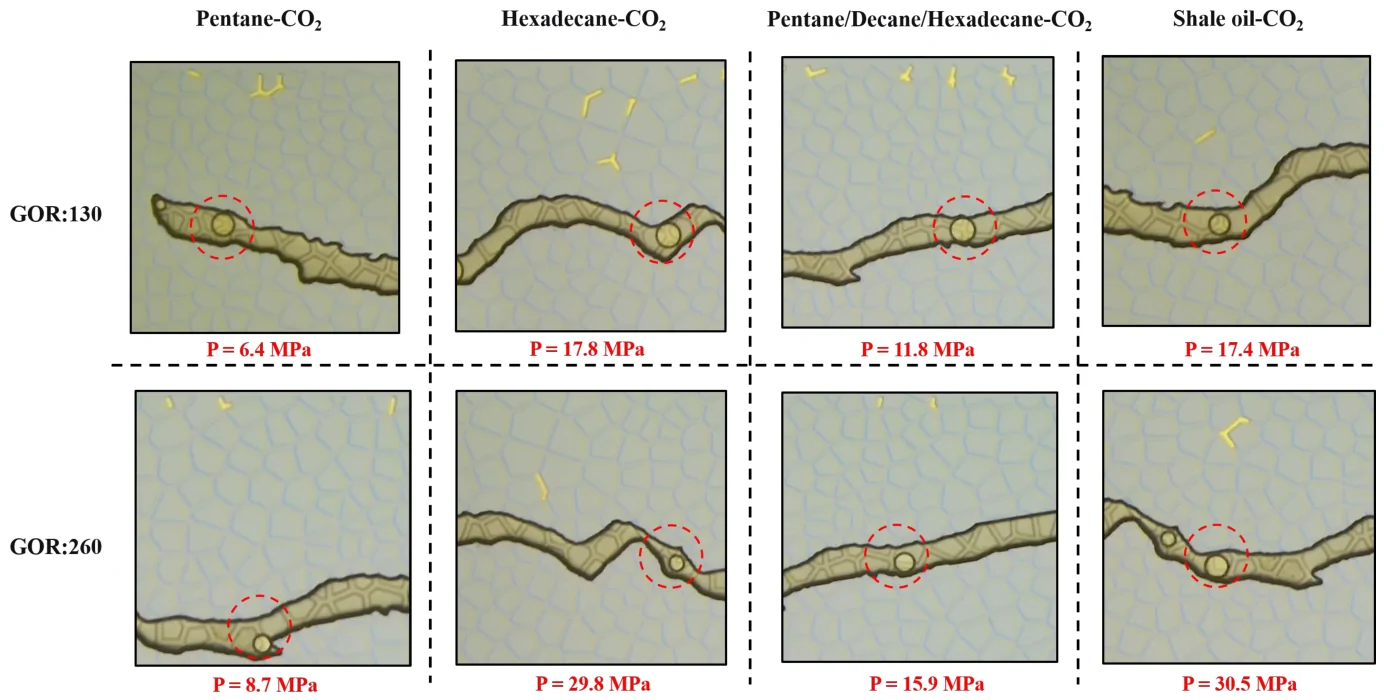
Bubble nucleation of CO_2_–oil systems in the coupled 10 nm–10 μm pore model at 105 °C.

The influence of pore-scale coupling was investigated by comparing the bubble-point behavior between the isolated 10 nm single-scale pore model and the coupled 10 μm–10 nm pore-network model. Figure 5 presents the bubble-point phenomena observed in the coupled multiscale pore system at 105 °C.

The bubble-point behavior of the coupled pore network differs significantly from that of the isolated 10 nm pore system. In the coupled model, the experimentally observed bubble points remain close to the equilibrium bubble-point prediction of the micrometer-scale pore domain, whereas the isolated 10 nm system exhibits pronounced downward deviations toward the metastable nucleation limit.

For GOR = 130, the measured bubble-point pressures in the coupled 10 μm–10 nm network are approximately 6.4, 17.8, 11.8, and 17.4 MPa for the pentane–CO_2_, hexadecane–CO_2_, pentane/decane/hexadecane–CO_2_, and shale oil–CO_2_ systems, respectively. At GOR = 260, the corresponding bubble-point pressures increase to approximately 8.7, 29.8, 15.9, and 30.5 MPa. Compared with the isolated 10 nm pore system, the coupled network exhibits much weaker deviation from the theoretical equilibrium phase boundary.

The quantitative comparison of the shale oil–CO_2_ system further demonstrates the effect of pore-scale coupling. According to Table 3, at GOR = 130, the relative deviation in the isolated 10 nm model decreases from 29.77% at 65 °C to 9.65% at 125 °C, whereas the coupled 10 μm–10 nm model exhibits deviations of only 2.43–1.29% over the same temperature range. At GOR = 260, the deviation decreases from 25.25–6.94% in the 10 nm model to only 1.24–0.29% in the coupled pore network. These results demonstrate that pore-scale coupling substantially weakens the apparent bubble-point shift caused by nanoscale confinement.

The comparison between the two pore configurations also reveals different relationships with the metastable nucleation limit. In the isolated 10 nm pore model, the experimental bubble points are located closer to the metastable nucleation limits, indicating that strong confinement promotes delayed vapor formation and enhances metastable behavior. In contrast, the experimental bubble points in the coupled 10 μm–10 nm network remain close to the equilibrium bubble-point curves of the micrometer-scale pore domain and are clearly separated from the metastable limits.

This behavior can be attributed to the different phase-transition environments provided by the two pore configurations. In isolated nanopores, the entire fluid system is subjected to strong surface interactions, restricted molecular mobility, and enhanced interfacial effects. These factors increase the resistance to vapor formation and shift the experimentally observed bubble point toward the metastable nucleation boundary. However, when nanopores are connected with larger pore domains, the micrometer-scale region provides a less confined environment for phase transition. Because the nucleation barrier is lower in the micrometer-scale region than in the strongly confined nanochannels, vapor nucleation preferentially occurs in the micrometer-scale domain during depressurization. Once a stable vapor phase forms in this region, the connected micro-nanopore network has already undergone an observable phase transition, without requiring independent nucleation to occur first within the 10 nm nanochannels. Consequently, the measured phase-transition pressure of the coupled system is governed primarily by the nucleation condition of the micrometer-scale region and therefore remains much closer to the micrometer-scale equilibrium bubble-point prediction. The weaker confinement effect and reduced interfacial restriction allow vapor formation to occur closer to equilibrium conditions.

Therefore, the bubble-point behavior of a connected multiscale pore system cannot be directly represented by the phase behavior of isolated nanopores. Although individual nanopores may exhibit significant nanoconfinement-induced metastable shifts, the presence of larger connected pore domains can substantially reduce the effective confinement effect observed at the reservoir scale. This finding highlights the importance of considering pore-scale connectivity when evaluating CO_2_–shale-oil phase behavior during CO_2_-enhanced oil recovery.

## 4. Conclusions

The results of this study demonstrate that the phase-transition behavior of CO_2_–oil systems under nanoscale confinement is governed by the combined effects of thermodynamic state, fluid composition, metastable nucleation, and pore-scale connectivity rather than by pore size alone. In isolated 10 nm pores, vapor formation is delayed relative to the equilibrium bubble-point boundary, causing the experimentally observed phase-transition pressure to shift toward the metastable nucleation limit. This deviation becomes weaker as the system approaches the critical region through increasing temperature or CO_2_ content, whereas heavier hydrocarbon systems exhibit a stronger tendency toward metastable vaporization. These findings indicate that the experimentally observed phase transition in nanopores reflects the coupled influence of equilibrium thermodynamics and nucleation constraints.

More importantly, the comparison between isolated 10 nm pores and the coupled 10 μm–10 nm pore network demonstrates that pore-scale connectivity can substantially weaken the apparent nanoconfinement effect. In the connected system, vapor nucleation preferentially occurs in the less-confined micrometer-scale region, causing the observable phase-transition pressure of the coupled network to remain close to the micrometer-scale equilibrium condition. Therefore, phase behavior measured or predicted for isolated nanopores should not be directly extrapolated to connected shale pore networks. Incorporating metastable nucleation and multiscale pore connectivity is essential for establishing a more physically representative description of CO_2_–shale-oil phase behavior and for improving phase-state evaluation during CO_2_-enhanced shale-oil recovery.

Several limitations should nevertheless be recognized. The present micro/nanofluidic models employ idealized pore geometries and cannot fully reproduce the complex pore-size distribution, surface heterogeneity, mineral composition, wettability, and connectivity of natural shale reservoirs. In addition, although the metastable limit model provides a reasonable interpretation of the experimentally observed bubble-point shifts, it is based on simplified nucleation theory and does not explicitly capture molecular-scale compositional redistribution, heterogeneous nucleation processes, or dynamic phase-transition pathways. Future studies should therefore incorporate more realistic heterogeneous pore networks, broader shale-oil compositions, and molecular-scale or pore-scale simulations to further clarify the coupled effects of nanoconfinement, metastability, and multiscale pore connectivity on CO_2_–shale-oil phase behavior.

## Figures and Tables

**Table 1:** Whole-oil carbon-number distribution of shale oil in terms of mass fraction.

Carbon Number	Mass Fraction (wt%)
C3	0.01
C4	0.02
C5	0.05
C6	0.17
C7	0.54
C8	1.26
C9	2.46
C10	4.26
C11	5.72
C12	6.27
C13	6.36
C14	6.47
C15	6.38
C16	5.94
C17	5.66
C18	5.36
C19	5.00
C20	4.53
C21	4.20
C22	3.88
C23	3.66
C24	3.23
C25	3.03
C26	2.58
C27	2.32
C28	1.98
C29	1.75
C30	1.46
C31	1.34
C32	1.04
C33	0.84
C34	0.69
C35	0.55
C36	0.42
C37	0.31
C38	0.26

**Table 2:** Deviations between experimental and theoretical bubble-point pressures for different fluid systems at GOR = 260 in the 10 nm pore model.

Components	Temperature/°C	Theoretical Bubble Point/MPa	Experimental Bubble Point/MPa	Deviation (%)
Pentane	65	6.5	6.2	4.10
	85	7.0	6.9	1.10
	105	Supercritical	Supercritical	/
	125	Supercritical	Supercritical	/
Hexadecane	65	15.2	9.1	40.63
	85	16.4	11.9	27.61
	105	17.4	14.3	18.00
	125	18.0	16.2	10.01
Pentane/Dodecane/Hexadecane	65	9.6	7.3	24.30
	85	11.2	9.5	15.09
	105	12.3	11.3	8.00
	125	12.9	12.7	1.57
Shale oil	65	14.8	11.1	25.25
	85	16.0	13.1	17.92
	105	17.0	15.0	11.93
	125	17.6	16.4	6.94

**Table 3:** Deviations between experimental and theoretical bubble-point pressures of the shale oil–CO_2_ system under different GORs, pore scales, and temperatures.

Condition	Temperature/°C	Theoretical Bubble Point/MPa	Experimental Bubble Point/MPa	Deviation (%)
Scale: 10 nm + 10 µm GOR:260	65	29.0	28.6	1.24
	85	29.9	29.5	1.19
	105	30.7	30.5	0.58
	125	31.4	31.3	0.29
Scale: 10 nm + 10 µm GOR:130	65	13.4	13.1	2.43
	85	15.7	15.4	1.84
	105	17.7	17.4	1.76
	125	19.3	19.1	1.29
Scale: 10 nm GOR:260	65	14.8	11.1	25.25
	85	16.0	13.1	17.92
	105	17.0	15.0	11.93
	125	17.6	16.4	6.94
Scale: 10 nm GOR:130	65	9.2	6.5	29.77
	85	10.5	8.2	21.51
	105	11.5	9.8	14.93
	125	12.2	11.1	9.65

## Data Availability

The original contributions presented in this study are included in the article/supplementary material. Further inquiries can be directed to the corresponding authors.

## Supplementary Materials

The following supporting information can be downloaded at: https://www.mdpi.com/article/10.3390/nano16191260/s1, Figure S1: AFM characterization of the 10 nm nanofluidic channel corresponding height profile across the nanochannel.
