# Supplementary material for: Bubble-Point Pressure Deviations and Metastable Nucleation in Nanoconfined CO2–Shale Oil Systems: Effects of Temperature, Composition, Gas–Oil Ratio, and Pore-Scale Coupling
Source: Nanomaterials (Basel). 2026 Oct 6;16(19):1260. doi: 10.3390/nano16191260 (PMC13667126; doi:10.3390/nano16191260)
Supplement: Supplementary file 1 [file nanomaterials-16-01260-s001.zip › nanomaterials-4587733-supplementary.pdf]

Supporting Information

Bubble-Point Pressure Deviations and Metastable Nucleation in Nanoconfined CO_2–Shale Oil Systems: Effects of Temperature, Composition, Gas–Oil Ratio, and Pore-Scale Coupling

Dongyan Qi [1,2], Jiawei Li [1,2,*], Qiang Liu [1,2], Shuang Wang [1,2], Suiyuan Cui [1,2], Yue Lang [1,2,*], Jiayi Zhao [3], Xiaozhe Liang [3] and Junjie Zhong [3]

1 Daqing Oilfield of China National Petroleum Cooperation, Daqing 163700, China

2 State Key Laboratory of Continental Shale Oil, Daqing 163712, China

3 State Key Laboratory of Deep Oil and Gas, School of Petroleum Engineering, China University of Petroleum (East China), Qingdao 266580, China

Keywords: shale oil; nanoconfinement; metastability; pore-scale connectivity; Peng–Robinson EOS; gas–oil ratio

Contents

AFM Characterization of the 10 nm Nanochannel

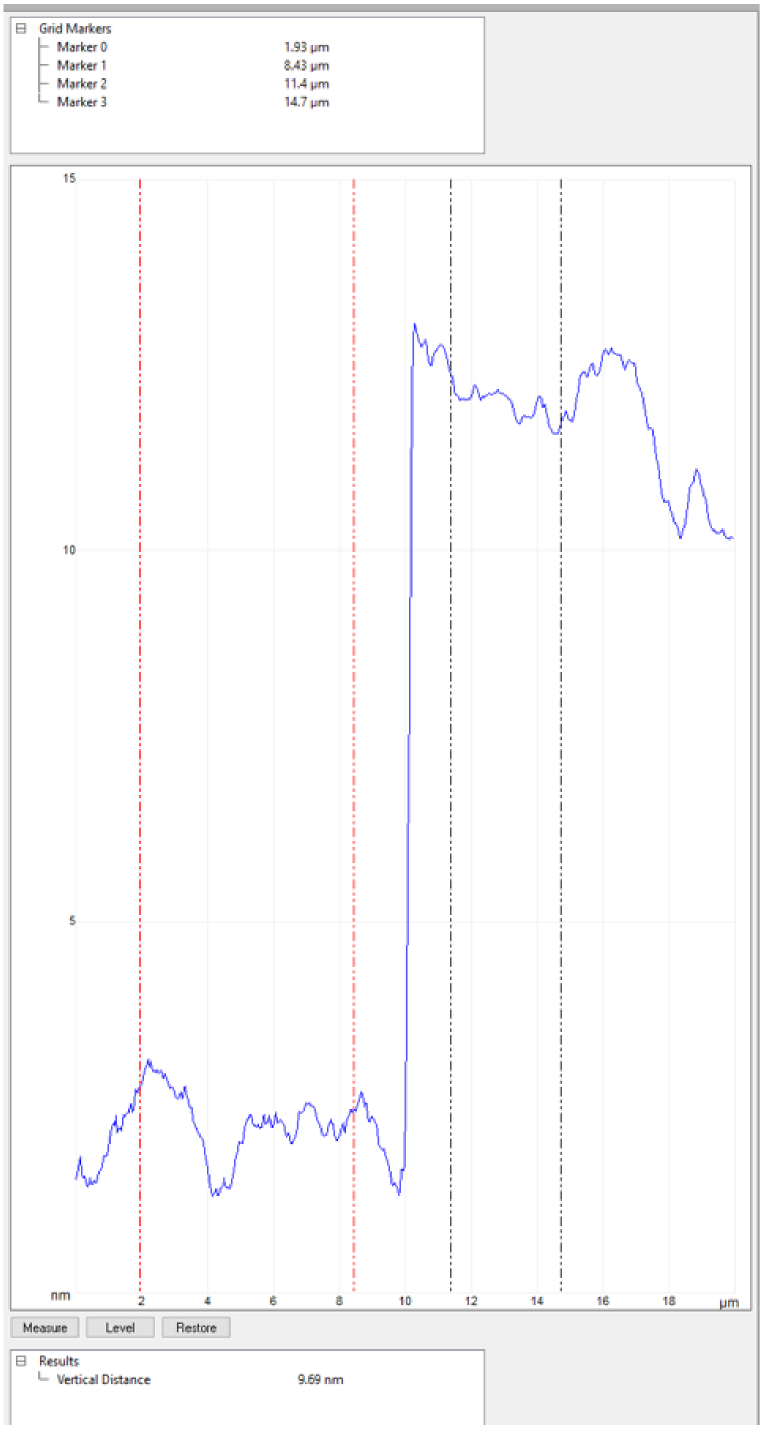

Figure S1. AFM characterization of the 10 nm nanofluidic channel corresponding height profile across the nanochannel.
